# Comparisons of home and daytime ambulatory blood pressure measurements

**DOI:** 10.1186/1471-2261-14-94

**Published:** 2014-08-01

**Authors:** Sigrun Chrubasik-Hausmann, Cosima Chrubasik, Brigitte Walz, Jürgen Schulte Mönting, Paul Erne

**Affiliations:** 1 Institute of Forensic Medicine, University of Freiburg, Albertstr. 9, Freiburg D 79104, Germany; 2 Abteilung Kardiologie des Kantonsspitals Luzern, CH 6000 Luzern 16, Luzern, Switzerland; 3 Zentrum für Labormedizin des Kantonsspitals Luzern, CH 6000 Luzern 16, Luzern, Switzerland; 4 Department of Medical Biometry and Informatics, University of Freiburg, Freiburg, Germany

**Keywords:** Ambulatory blood pressure measurements, Home-based self measurements

## Abstract

**Background:**

Home (HBPM) and ambulatory (ABPM) blood pressure measurements have their advantages and disadvantages in diagnosing and managing hypertension. We studied HBPMs and ABPMs in volunteers taking part in a survey.

**Methods:**

Of 366 respondents, 270 provided a total of 5997 triplicate HBPMs **(*Part 1*)**; 175 also provided data on ABPMs, of which the measurements obtained between 6 am and 10 pm were used in this study ***(Part 2*)**.

**Results:**

***Part 1***, When all 5997 triplicate HPPMs were considered, 1st readings tended to be significantly higher than those of the 2nd and 3^rd^ for both, systolic and diastolic pressures, but when the consideration was restricted to the very first triplicate of each of the 270 subjects, this was true only for systolic HBPM. ***Part 2***, The ABPMs tended to have a wider range than the corresponding HBPMs, and to be distributed towards higher values. Of the non-parametric indices of (ABPM - corresponding HBPM), (First Quartile, Median, Third Quartile and Maximim) all but the minima had positive values.

**Conclusions:**

In triplicate HBPMs, the first measurement is usually but not always the highest. Increasing the number of triplicates provided by each subject increases the chance of discriminating between measurements in the triplicates. ABPMs tended to be higher than the corresponding HBPMs.

## Background

There is little point nowadays in simply classifying people as “hypertensive” or “non-hypertensive” purely on the basis of one blood pressure measurement - no matter by what means or how confidently it may have been made. For some applications (for example, in monitoring or researching the effect of antihypertensive medication on blood pressure) it is important to be confident about baselines and the changes that may occur with medication. For other applications such as assessing cardiovascular risk, additional factors are at least as important as the blood pressure measurement and choice of the means by which blood pressure is measured may be less critical.

Home and ambulatory blood pressure measurements (HBPMs, ABPMs) have their advantages and disadvantages in diagnosing and managing hypertension. Subjects who are taught to use the devices can carry out HBPMs, away from many of the disturbances present in clinical settings, but the time and effort required of the subject place a limit on the number and timing of the measurements that can be made. This is irrespective of recommendations made in various guidelines [1-4]. ABPM was establied before HBPM. It can be carried out with little effort on the part of the subject other than to try not to disturb the measurement and can be repeated at relatively short intervals but resource limitations mean that ABPM is usually restricted to no more than 24 hours at a time. ABPM has been advocated as a gold standard [5] for determining whether a subject needs to be started on antihypertensive management or to have the management changed. However, it is less feasible for monitoring the time-course of any response to that treatment.

We have used data from a blood pressure screening program in order to examine:

(i) The discriminating power offered by more numerous HBPMs for distinguishing the first measurement in a triplicate from the second and third;

(ii) The difference between ABPM and HBPM in the same subject.

## Methods

The protocol for this single-centre surveillance study was approved by the Committee for Human Ethics of the Kanton Luzern/Switzerland. The study was advertised locally by word of mouth and by flyers distributed in the hospital, inviting would-be participants to contact the Department “Arbeitsmedizinischer Dienst” of the Kantonsspital Luzern. Exclusion criteria included a need for immediate antihypertensive treatment, angina pectoris, atrial arrhythmia, vitium cordis with haemodynaemic impairment, chronic renal or liver diseases and actual or contemplated pregnancy. After written informed consent and assessments of baseline conditions with a standardized questionnaire, blood pressure was measured by the staff using a boso-medicus PC oscillometric device validated according to the AAMI and BHS protocol (grading A/A), Bosch + Sohn GmbH u. Co KG, Jungingen (data of the validations are placed on http://www.uniklinik-freiburg.de/rechtsmedizin/forschung/phytomedizin.html, details to original articles).

(i) Participants were then shown how to measure their own blood pressure correctly. Standard size 12 cuffs were used except in patients whose arm circumference exceeded 33 cm, for whom size 15 cuffs were used. They were asked to try to undertake HBPMs three times a day (morning, lunch time, evening) for the surveillance period. Each measurement was to be made in triplicate, 2 minutes apart, after resting seated for at least 10 minutes, and at least 2 hours after any coffee or smoking. Heart rate was recorded simultaneously. Seven to 10 days later, the HBPM profile was printed out.

(ii) Those participants whose HBPM exceeded 125/80 mmHg on average over the surveillance period were invited to participate in a comparison of ABPM and HBPM. The ABPMs were made on the same arm as for the HBPM, using boso TM-2430 PC oscillometric devices. These devices, graded A/A, (best category for systolic and diastolic measures), were validated according to the protocol of the Advancement of Medical Instrumentation and British Hypertension Societies. ABPMs were made every 15 min from 6 am to 10 pm and participants were asked to try to keep the arm still during each measurement. (ABPMs were also made every 30 min from 10 pm to 6 am, but these are not considered in this paper). After the 24 hours of recording, the participant returned to the “Arbeitsmedizinischer Dienst” with a record of his or her activities of the day and the quality of the night’s rest. The data were transferred to the software boso-profilmanager 2 (descriptions of the handling of the devices and data transfer are placed on the above mentioned website).

Statistical analysis was carried out using the UNIVARIATE, NPAR1WAY and CORR procedures in SAS 9.2, and the figures were plotted using Microsoft Excel.

For a subsidiary investigation into whether there was a gender-related difference in median HBPM in the 347 patients who supplied an adequate number of readings, we used the Multiple Regression procedure in Microsoft Excel to regress participants’ ages, weights, heights and a dummy variable for gender against the median HBPM. We also did it for the 175 participants who were selected by the blood pressure criterion of 125/85 for the comparison between HBPM and ABPM.

## Results

### Part 1: Triplicate home blood pressure measurements

**Figure 1:**
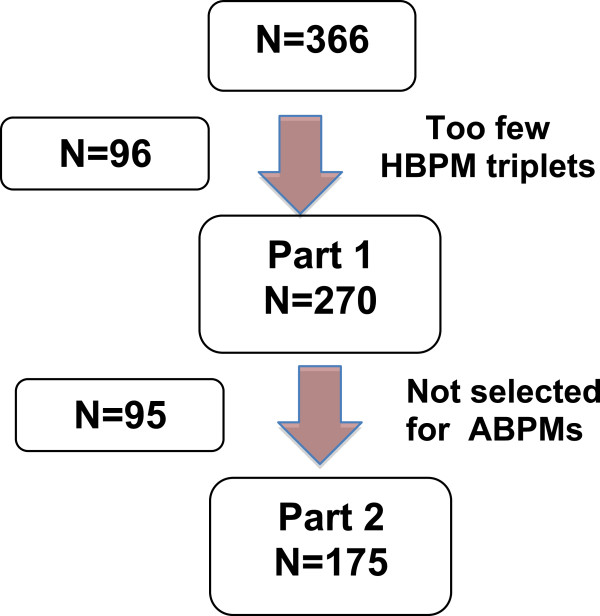
Flow chart of the in- and excluded participants.

The characteristics recorded for the 366 consecutively recruited participants are summarised in Table 1. Of these, 96 were excluded because they failed to follow instructions to make 3 triplicate HBPMs per day and/or contributed fewer than 27 measurements in total (Figure 1). The remaining 270 participants provided 5997 triplicates, − an average of 22.2 triplicates (out of a maximum of 30) per participant. Table 2 indicates the distributions of values of systolic and diastolic HBPM and heart rate (HR) for the 1st, 2nd and 3rd values in the total of 5997 triplicates. Table 3 indicates the corresponding distributions for the 1st triplicate contributed by each of the 270 participants. Friedman testing on the data from all triplicates (Table 2 indicated differences, significant at the p < 0.001 level, between the 1st and second and the 1st and 3rd readings. The general rule was that 1st systolic and diastolic HBPM and HRs were greater than the 2nd and 3rd values, but there was sufficient variability for the general rule to be inapplicable in about one third of the triplicates.

**Table 1:** The characteristics recorded from the 366 volunteers who responded to the publicity about the study

	***Mean***	***SD***
*Age men (years)*	*52*	*(13)*
*Age women (years)*	*53*	*(14)*
*BMI men (kg/m*^2^*)*	*27*	*(5)*
*BMI women (kg/m*^*2*^*)*	*25*	*(5)*
Numbers and percentages of patients:	N	(%)
Male: female:	179:187	(49:51)
With triplicate measurements	270	(74)
With mean home blood pressure measurement > 125/80	200	(54)
With family history of cardiovascular disease:	107	(29)
Current smokers	150	(41)
Sports activities > 20 min/day:	119	(33)
Previous myocardial infarction:	8	(2)
Angina pectoris:	6	(2)
Previous bypass surgery:	2	(1)
Previous percutaneous transluminal coronary angioplasty	9	(2)
Previous stroke	8	(2)
Subjected to salt restriction:	80	(22)
Numbers taking antihypertensive medications	97	(27)
	*Mean*	*SD*
*Dose of antihypertensive agentss expressed as equivalents:*	*1.8*	*(1.5)*
*1 equivalent = hydrochloro thiazide 25 mg = enalapril 5 mg = lorsartan 50 mg*		
*= metoprolol 100 mg = amlodipine 5 mg = isosorbide dinitrite 40 mg*		
*= doxazozine 2 mg = clonidine 15 mg*		
Numbers taking medications for other reasons		
Contraception:	14	(4)
Menopausal symptoms	20	(5)
Insulin:	4	(1)
Anti-diabetics:	13	(4)
Low-dose aspirin	31	(8)
Magnesium supplementation:	72	(20)
Anti-inflammatory drugs (NSAIDs):	15	(4)
	*Mean*	*SD*
*Dose of NSAIDs expressed as diclofenac equivalents:*	94	(61)
*diclofencac 100 mg = metamizol 1330 mg = acetylsalicylic acid 1300 mg*		
*= ibuprofen 800 mg = naproxen550 mg = propyphenazone 250 mg*		
*= acemetacine 120 mg = ketoprofen 66.6 mg = indomethacin 50 mg*		
*= piroxicam 6.66 mg.*		

**Table 2:** HBPM (mmHg) and heart rate (beats/min) of all available triplicates (n = 5997, average of 22.2 sets of triplicates per subject)

**Variable**	**Mean**	**SD**	**Minimum**	**Maximum**
Systolic_1st	126	17	55	234
Systolic_2nd	123	16	76	200
Systolic_3rd	122	16	73	243
Diastolic_1st	78	12	28	203
Diastolic_2nd	77	11	39	165
Diastolic_3rd	77	11	37	232
Heart rate_1st	70	12	40	128
Heart rate_2nd	69	11	39	125
Heart rate_3rd	69	11	38	135

**Table 3:** HBPM (mmHg) and heart rate (beats/min) of the very first triplicate (first morning triplicate) for each subject (n = 270)

**Variable**	**Mean**	**SD**	**Minimum**	**Maximum**
Systolic_1st	127	19	90	234
Systolic_2nd	123	17	87	195
Systolic_3rd	123	16	87	184
Diastolic_1st	80	14	41	203
Diastolic_2nd	79	11	44	128
Diastolic_3rd	79	11	52	128
Heart rate_1st	68	10	44	106
Heart rate_2nd	66	10	46	105
Heart rate_3rd	66	10	41	104

When all participants were considered, maleness was associated with a significantly higher median HBPM than femaleness, even when age, height and weight were allowed for, but this was not so for the 175 patients who were selected for ABPMs. In the sample of 347 participants patients, the ratio of males to females was 1.012:1. In the sample of 175 selected by the blood pressure criterion, the ratio was 1.1875 to 1. Details are given on the webpage.

### Part 2: Comparison of ambulatory and home blood pressure measurements

**Figure 2:**
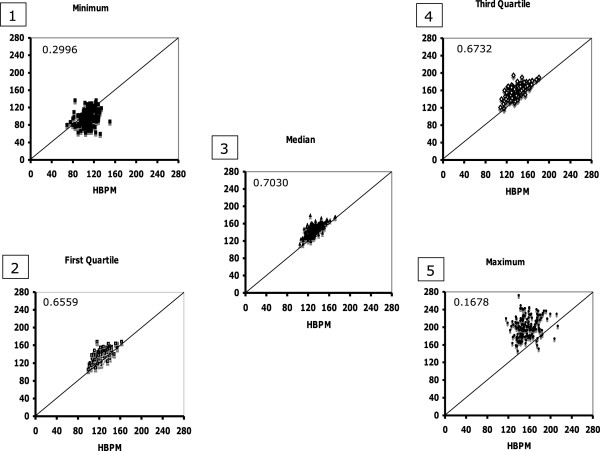
**Participant-by participant plots of the indices of distribution of HBPM against the corresponding indices of distribution of ABPM.** The diagonal line is the line of equality.

Of the participants whose average HBPM exceeded 125/80, 175 had reasonably complete BPM sets with a mean of 45 systolic HBPM daytime readings (15 triplicates) per participant and 63 systolic ABPM daytime readings (Figure 1).The distributions of the HBPMs over up to 10 days and of ABPMs over up to 18 hours were compared in terms of their minimum, 1st, 2nd and 3rd quartile, and maximum values. The five indices of ABPM were plotted against the corresponding indices of HBPM in Figure 2. The minima are scattered mostly below the line of equality, but without much adherence to its slope. The first quartile, median and third quartile adhere quite well to the slope of the line of equality, the first quartile being closest to it and the median and third quartile being displaced progressively above it. The maxima are scattered, mostly above the line of equality, but without much adherence to it. The Pearson correlation coefficients show a modest correlation between the respective first quartiles, medians and third quartiles, but weak correlation between the minima and maxima. The dotted rectangles enclose the ranges ABPM (vertical) and HBPM (horizintal) observations in each plot. They are not strikingly different between plots and between ABPM and HBPM.

Further data on the distributions of ABPM and HBPM in terms of the median values of their five indices of distribution (Minimum, 1st Quartile, Median, 3rd Quartile and Maximum) are placed on the above mentioned website.

### Differences between ABPM and HBPM

**Figure 3:**
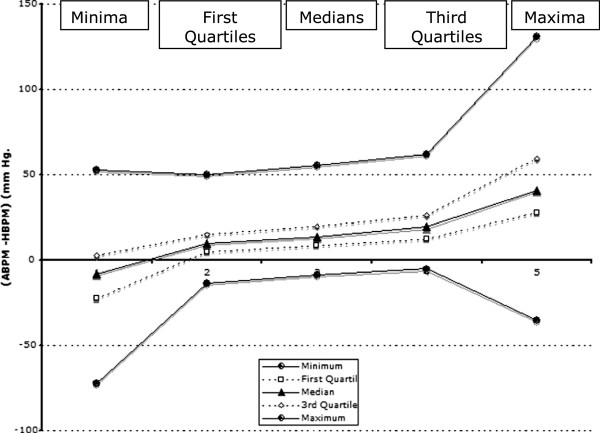
Indices of distribution of the indices of distribution of the values ABPM - HBPM.

In each sub-plot of Figure 2, the vertical distance between each plotting symbol and the line of equality is the difference between ABPM and HBPM (ABPM – HBPM) for the corresponding index of distribution for that participant. Within each dotted rectangle in each sub-plot there will be a set of indices of distribution (Minimum, 1st Quartile, Median and 3rd Quartile, Maximum) for ABPM minus HBPM. These are shown in Figure 3. The vertical distance between the uppermost and lowermost lines shows the span between the maxima and minima of the respective indices of distribution. It is of the order of 70 mm Hg for the 1st Quartiles, Medians and 3rd Quartiles, but increased to about 130 mm Hg. For the 1st Minima and about 170 mm Hg for the Maxima. The Minima tended to be distributed towards negative values of (ABPM-HBPM), but towards progressively more positive values for the Medians, 3rd Quartiles and Maxima.

## Discussion

This was a purely observational study with no intervention and no prospective hypothesis to test. Any references to “statistical significance” are notional rather than definitive.

### Part 1: Triplicate home blood pressure measurements

There is disagreement between guidelines for self-measured BPMs at home: (i) the European Society of Hypertension [1] and the 2012 Canadian Hypertension Education Program [2] recommend duplicate HBPMs in the morning and evening; (ii) the American Society of Hypertension [3] recommends triplicate HBPMs [4]; (iii) the Japanese Society of Hypertension recommends at least one HBPM [5]. As reported here and elsewhere [6,7], the 1st HPPM in a triplicate tends to be higher than the 2nd and 3rd. The differences are quite small. The statistical significances attached to the values in Table 2 and Table 3 arise more from their consistency than from their size. They amount, on average, to 3 – 4 mmHg for systolic BP, 1 mmHg for diastolic and 1 – 2 mmHg for the heart rate. These amount respectively to about 18–25%, 9% and 9–10% of the associated standard deviations. Inverting the simple power calculations to obtain numbers of triplicates needed to have 80% power to attach significance at the 5% level gives 268 – 475 triplicates for systolic BP, 2077 – 2453 for diastolic, and 393–2077 for heart rate. The repeated measures element of the Friedman test that was applied would have provided somewhat more discriminating power because it separated differences in measurement within triplicate from differences between triplicates. However the extra discriminating power was not sufficient for the significance of the differences in diastolic pressure and heart rate that were seen for the 5997 triplicates represented in Table 2 to be apparent also in the 270 triplicates in Table 3.

Nonetheless, the differences between triplicates have been sufficient in at least one study for the diagnosis of “hypertension” to be made more frequently with the first than with the mean of the 2nd and 3rd [8]. Consequently, they may have some effect on selecting patients for inclusion in trials of antihypertensive medication, or conceivably commencement or modification of anti-hypertensive therapy. However it is hardly conceivable that patients would be started on such therapy solely on a BPM if that measurement was so close to the chosen criterion that the differences in measurement in a triplicate would be crucial. The decision would surely have to be based on a more comprehensive assessment of risk.

Some of the guidelines recommend discarding the higher HBPMs that are often measured initially, but this refinement had no impact in explanatory modelling of cardiovascular risk [9] or organ damage [10]. In keeping with the greater precision evident in Table 2 than in Table 3, one would expect the precision in estimating CVR to increase with the number of measurements contributing to the estimate of BP, though possibly only up to a point. For example, Niiranen et al. [10] found that the most precise predictions were achieved by including all values of duplicates measured twice per day over 7 days, though the measurements made over the first 3 days were more influential than those made later.

Timing of the HBPMs and choice of subject may also be important. For example, in a mixture of normotensives and hypertensive patients seen in the clinic, evening HBPMs predicted stroke more reliably than casual HBPM regardless of the number of measurements [11]. On the other hand, morning HBPMs predicted stroke more reliably than evening HBPMs in patients on hypertensive medication but not in normotensive patients [12]. Likewise, the *variability* in HBPM had an additional predictive power beyond mean systolic HBPM only in hypertensive patients [13].

Kawabe et al. [14] reported that the 1st systolic HBPM in morning triplicates had a larger coefficient of variation (CV) than the 2nd and was probably affected by the subject’s gender and smoking habits. In our Tables 2 and 3, the standard deviations did not seem very different between measurements in the triplicates.

The gender differences obtained in our small sample were consistent with those in the review by Reckelhoff [15].

### Part 2: Comparison of ambulatory and home blood pressure measurements

ABPM was developed before HBPM and remains more copiously documented. It can also identify a larger number of features than either clinic or HBPM (e.g. “white coat hypertension”, night-time ABPM, the night-to-day BP ratio and “dipping” status - which are significant predictors of outcome [16]). It has therefore acquired ”gold standard” status though this does not necessarily mean that average or median ABPM is, by itself, the most accurate indicator of true normo-/hypertensive status. Unlike ABPMs, *self-measured* HBPMs can only be obtained while the patient is awake. The best agreement between ABPM and HBPM appeared to be in the morning between the first HBPM of the day and the mean of the 4 observations taken in the second hour after the patient had wakened [17]. We have restricted our comparison to *daytime* ABPMs and HBPMs.

A systematic review including six comparisons of HBPM and ABPM found that blood pressures over the criterion of 135/85 mmHg were obtained more frequently overall with HBPMs [5]. However, in the three studies with the largest numbers of HBPMs (29 to 56), the average ABPMs were higher than the HBPMs, to a lesser or greater degree. In one [18], the difference was similar to that found in our previous study [19] with a similar number of HBPM measurements.Our present results were from a data set that included an average of 45 HBPM and 63 ABPM measurements per participant. The comparison are in terms of the indices of distribution of the sets of observations of HBPM and ABPM in each subject across a set of subjects, as illustrated in our Figure 2 and Figure 3. For the 1st, 2nd and 3rd quartiles: (i) increases in individual ABPMs between patients tended to correlate reasonably well with increases of the corresponding HBPMs (r close to 0.7 in plots 2, 3 and 4 in Figure 2); (ii) ABPM tended to be greater than the corresponding HBPM by amounts that increased with increase in BP (by 10 mm Hg for the median of the 1st quartiles, 14 mm Hg for the 2nd and 19.5 mm Hg for the 3rd, Figure 3). At the extremes of the distributions: (i) there was less correspondence between the increases in ABPM and HBPM (r less than 0.3 for plots 1 and 5 in Figure 2); (ii) the spans of the distributions of the minima and maxima were wider, and the minima of the distributions of the 1st, 2nd 3rd quartiles were at negative values of (ABPM – HBPM, Figure 3).

It is a plausible conjecture that the behaviour of the measurements at the extremes of their range may be partly due to movement-related measurement artefacts in the ABPMs: the subject may be less inclined to interrupt his or her activity every 15 minutes for the duration of each ABPM whereas he or she might be more likely to stop and be settled for the HBPMs, at least by the time the 2nd and 3rd measurement in each triplicate has been made. The relative symmetry of both distributions in our Figure 2 about their medians is consistent with the observation of Lijarcio [20] who demonstrated, that for HBPM, the medians and means are more or less interchangeable – which was corroborated by detailed comparisons within our data set. The observations in Figure 2 are consistent with our previous observations [20] that, with duplicate HBPMs 3 times per day for a week, the coefficient of variation of the HBPMs was smaller than that of daytime ABPM obtained between 7 am and 10 pm on a single day, (4.2% compared with 5.5%).

Irrespective of whether it is the ABPM or HBPM that gives a “truer” picture of whether or not a subject should be labelled “hypertensive” at a particular time, we have argued previously on pragmatic grounds [19] that the smaller CVs associated with HBPM should make them more useful for detecting sooner and more reliably if, when, and how quickly a change in the subject’s underlying blood pressure has taken place, whether in response to institution or modification of treatment, or to other intercurrent influences. A further pragmatic advantage of HBPMs for *longer-*term monitoring is that because of resource limitations, facilities for ABPMs are usually only available over a *shorter* time and are more intrusive on activities of daily living.

## Conclusions

(1) Numbers of observations do matter. Recommendations for types and numbers of BPM should take into account the stringency required for the purpose to which the BPMs are being put.

(2) A more effective harmonization of guidelines is desirable when studying the effects of interventions on blood pressure, not least if those studies are to be included in systematic reviews and meta-analyses

(3) Resource limitations, and the time-scale required for the measurements have an important bearing on which measurement technique to use and how many measurements to make.

## Competing interests

The authors declare that they have no competing interests.

## Authors’ contributions

All authors have made substantial contributions. Conception: SC-H, PE. Design: SC-H, PE. Acquisition of data: CC (†), BW. Statistical analysis: JS-M. Interpretation of data: SC-H, PE and AB (acknowledged). SC-H wrote the draft which was revised critically for important intellectual content by all authors except CC who died in a tragic accident. All authors except CC have given final approval of the version to be published. Each author should have participated sufficiently in the work to take public responsibility for appropriate portions of the content. All authors read and approved the final manuscript.

## Pre-publication history

The pre-publication history for this paper can be accessed here:

http://www.biomedcentral.com/1471-2261/14/94/prepub
